# Successful use of pazopanib for treatment of refractory metastatic hemangiopericytoma

**DOI:** 10.1186/2045-3329-4-13

**Published:** 2014-09-24

**Authors:** Su Jin Lee, Seung Tae Kim, Se Hoon Park, Yoon La Choi, Jae Berm Park, Sung-Joo Kim, Jeeyun Lee

**Affiliations:** 1 Division of Hematology-Oncology, Department of Medicine, Samsung Medical Center, Sungkyunkwan University School of Medicine, 50 Irwon-dong Gangnam-gu, Seoul 135-710, Korea; 2 Department of Pathology, Samsung Medical Center, Sungkyunkwan University School of Medicine, Seoul, Korea; 3 Department of Surgery, Samsung Medical Center, Sungkyunkwan University School of Medicine, Seoul, Korea

**Keywords:** Hemangiopericytoma, Pazopanib, Anti-angiogenic agent

## Abstract

Hemangiopericytoma is a rare disease entity of soft-tissue sarcoma (STS) that can be cured with surgical resection. In cases of inoperable recurrence or metastasis, palliative chemotherapy is indicated, though there is currently no approved chemotherapy regimen. Therefore new treatment regimens are needed.

We describe three cases of metastatic hemangiopericytoma. In the first case, five lines of chemotherapeutic agents were used unsuccessfully in a patient with a 12-year history of metastatic hemangiopericytoma. After one cycle of pazopanib therapy, however, chest radiography showed a decrease in tumor volume of more than 30%. A marked decrease in FDG uptake on PET CT was also noted, and the patient is now on her 5^th^ month of pazopanib therapy. The second case is a patient with a brain hemangiopericytoma with multiple liver, lung, and bone metastases. Pazopanib induced radiologic stabilization of metastatic disease over the course of 8 months. The third case is a patient with a retroperitoneal hemangiopericytoma with pleural and peri-renal metastases. For more than 8 months, he has exhibited stable disease with pazopanib treatment.

Pazopanib may be useful for treatment of metastatic hemangiopericytoma, though further studies are needed to confirm the efficacy of this medication and to investigate its molecular mechanism of action.

## Introduction

Hemangiopericytoma was first described in 1942 by Stout and Murray [1] as a distinct soft tissue neoplasm, presumably of pericyte origin, exhibiting a characteristic well-developed “staghorn” branching vascular pattern. However, hemangiopericytoma has been reclassified as a fibroblastic neoplasm similar to a solitary fibrous tumor (SFT) [2,3]. It typically affects adults aged 20–70 years, with a median age in the 40s [4,5]. Common sites of involvement include the lower extremities, retroperitoneum/pelvis, lung/pleura, and meninges, though it may be found in virtually any part of the body [4,6].

Several patients with hemangiopericytoma have been successfully managed with surgical resection. However, approximately 15-20% of patients develop local recurrence or distant metastasis [4], and additional resections are not always feasible. The most common sites of metastasis are the lung, bone, and liver. Although there is no standard treatment for patients with advanced disease that is unresectable, anthracycline and ifosfamide-based chemotherapies are widely used.

Here, we describe three patients with metastatic hemangiopericytomas who were treated with pazopanib. The first patient achieved a partial response after one month of pazopanib therapy, while the second and third patients had stable disease over the course of 8 months of treatment based on RECIST v1.1 [7].

## Case presentation

**Figure 1:**
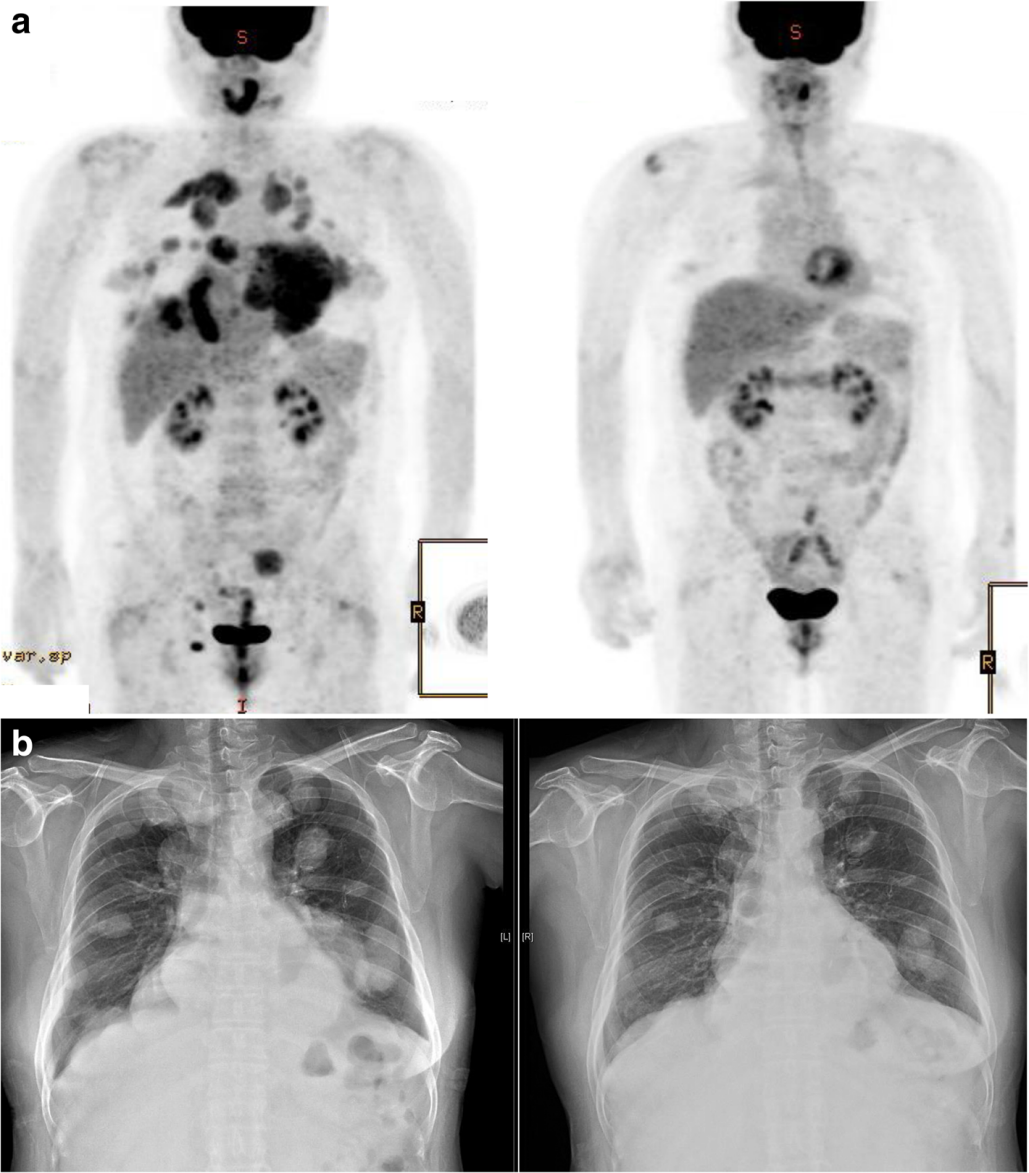
**Response to pazopanib therapy in case 1. a**. PET-CT at baseline and after 3 months of pazopanib treatment. **b**. Chest radiograph at baseline and after 4.5 months of pazopanib treatment.

The first patient was a 49-year-old female diagnosed in 2001 with multiple lung metastases from a hemangiopericytoma of an unknown primary site. After 6 cycles of first-line chemotherapy (doxorubicin and ifosfamide), her tumor response was classified as ‘stable disease’, and metastatectomy of the lung was performed in both 2002 and 2004. In 2005, a CT-scan showed multiple lung metastases that were inoperable. Due to the slow progression of her disease, she did not receive palliative chemotherapy until 2009. Beginning in 2009, she had nine cycles of second line chemotherapy (docetaxel/gemcitabine), twenty cycles of third line chemotherapy (everolimus), one cycle of fourth line chemotherapy (dacarbazine/cisplatin), and eight cycles of fifth line chemotherapy (ifosfamide, etoposide, cisplatin). In January 2014, the patient presented with pulmonary progression of disease, and was started on pazopanib treatment (800 mg daily). After one cycle of pazopanib, chest radiography showed a tumor response and the patient reported symptomatic improvement. After three months of pazopanib treatment, PET-CT and chest CT showed a partial response with more than a 50% decrease in tumor volume and a marked decrease in FDG uptake (maximum SUV for pelvic wall mass from 5.2 to 1.5 and right upper lung mass from 5.9 to 2.0) (Figure 1). She is now on her 5^th^ month of pazopanib therapy, and continues to maintain a ‘partial response’.

The second patient was a 52-year-old male who was diagnosed with a brain hemangiopericytoma after craniotomy and tumor removal in 2003. The patient experienced several post-operative recurrences in the brain that were treated with gamma-knife surgery. In 2011, recurrence was detected in the liver, and he underwent left hemihepatectomy. Six months later, another recurrence in the liver was treated with radiofrequency ablation. In July 2012, he developed metastases to the lung, liver, and pancreas, and first-line chemotherapy was administered (ifosfamide/etoposide/cisplatin). After 4 cycles of chemotherapy, a CT scan showed ‘stable disease’, but the patient was lost to follow-up. Several months later, the patient developed back pain, and metastasis to the spine at L1 was identified and treated with stereotactic radiation therapy. Pazopanib 800 mg daily was started in August 2013, and he has exhibited stable disease of lung, liver and pancreas over the course of 9 months of treatment. Except grade 1 diarrhea, there was no adverse event.

A third patient was a 54-year-old male patient with a retroperitoneal hemangiopericytoma that was diagnosed after resection of the mass in 1998. Adjuvant radiotherapy was done after surgery. Fifteen years later, multiple pleural, peri-renal and abdominal wall metastases were confirmed on a biopsy of one of the pleural masses. Palliative first line chemotherapy (ifosfamide/etoposide/cisplatin) was administered for 4 cycles, and his tumor response was classified as ‘stable disease’. Second line chemotherapy (docetaxel/gemcitabine) was then administered, and again the patient was classified as having ‘stable disease’. After 6 cycles of chemotherapy, however, his disease especially in lung progressed. He has since been taking third line pazopanib for eight months, and has again achieved ‘stable disease’. He was also tolerable to pazopanib 800 mg daily dose and no dose reduction was needed.

## Discussion

Although anthracycline-based therapies are frequently used, limited data is available regarding the efficacy of systemic chemotherapy in cases of advanced hemangiopericytoma. A few cases of hemangiopericytomas that have responded to chemotherapeutic agents have been reported, but no systematic reviews or clinical trials to date have identified an effective systemic treatment regimen, and the effects of these agents on PFS and OS remain uncertain [4,8-10].

The rich vascular characteristics of hemangiopericytomas and IHC expression of VEGF in these tumor cells [11-13] have prompted clinicians to try antiangiogenic therapies. Case reports have suggested that interferon-α may play a role in achieving disease stabilization in patients with hemangiopericytoma [9,14]. Park *et al*. reported retrospective data on use of temozolomide and bevacizumab in 14 patients with hemangiopericytoma/malignant solitary fibrous tumor in 2011 [15]. Eleven of 14 patients (79%) demonstrated Choi PR, and two patients achieved stable disease. The best response observed, as measured in RECIST, showed one PR and 13 cases of stable disease. The precise mechanisms by which temozolomide and bevacizumab exert their effects on hemangiopericytoma are not clear [5], but this regimen showed promising efficacy in that study.

Several case reports and retrospective studies suggested that imatinib [16,17], sorafenib [18], and sunitinib [18-21] could achieve long-lasting stable disease, although they did not induce a RECIST response. De Pas *et al*. reported a case in which clinical benefit was observed for 21-months with use of imatinib, which induced expression of PDGFR-α and PEGFR-ß [17].

Pazopanib (GlaxoSmithKline, Stevenage, UK) is a novel oral multi-targeted tyrosine kinase inhibitor with a wide range of activities that are mediated through VEGFR types 1, 2, and 3, platelet-derived growth factor receptors α and ß, and stem-cell factor receptor (c-Kit) [22,23]. A phase III trial of pazopanib showed PFS gain when compared with placebo in patients with metastatic non-adipocytic soft-tissue sarcoma (STS) after standard chemotherapy [24]. Based on that study, pazopanib was approved and has been used in most cases of relapsed or refractory STS. However, the efficacy of pazopanib in specific types of STS, especially hemangiopericytoma, is unknown. A recent subgroup analysis reported that four of seven patients with solitary fibrous tumors exhibited longer-term PFS and OS with pazopanib treatment [25,26].

The first case discussed is the first report of a RECIST response with use of pazopanib in patients with hemangiopericytoma. The precise mechanism by which pazopanib exerts its effect on hemangiopericytomas is poorly understood and requires further investigation.

## Conclusion

These three cases demonstrate that pazopanib may have clinical efficacy in patients with metastatic hemangiopericytoma, and thus pazopanib should be considered as potential treatment option for these patients.

## Consent

Written informed consent was obtained from the patients for the publication of this report and any accompanying images.

## Competing interest

The authors declare that they have no competing interest.

## Authors’ contribution

JL conceived and designed the study and supervised the research; SJL drafted the manuscript; STK, SHP,Y.LC, JBP, S-JST. STK provided comments on the manuscript. All authors read and approved the final manuscript.
